# Abnormal Maternal Body Mass Index and Customized Fetal Weight Charts: Improving the Identification of Small for Gestational Age Fetuses and Newborns

**DOI:** 10.3390/nu15030587

**Published:** 2023-01-22

**Authors:** Nieves Luisa González González, Enrique González Dávila, Agustina González Martín, Marina Armas, Laura Tascón, Alba Farras, Teresa Higueras, Manel Mendoza, Elena Carreras, María Goya

**Affiliations:** 1 Department of Obstetrics and Gynecology, University of La Laguna, Hospital Universitario de Canarias, 38200 Tenerife, Spain; 2 Department of Mathematics, Statistics and Operations Research, IMAULL, University of La Laguna, 38200 Tenerife, Spain; 3 Department of Obstetrics and Gynecology, Hospital Universitario Ntra Sra de Candenlaria, 38200 Tenerife, Spain; 4 Department of Pediatrics, Evangelisches Krakenhaus König Elisabeth Herzberge, 10365 Berlin, Germany; 5 Maternal-Fetal Medicine Unit, Department of Obstetrics, Hospital Universitari Vall d’Hebron, Universitat Autónoma de Barcelona, Pg. de la Vall d'Hebron, 119, 08035 Barcelona, Spain

**Keywords:** customized growth charts, fetal weight, newborn weight, maternal body mass index, obesity, thinness, small for gestational age, birthweight, newborn weight, perinatal outcomes

## Abstract

Background: Obesity and thinness are serious diseases, but cases with abnormal maternal weight have not been excluded from the calculations in the construction of customized fetal growth curves (CCs). Method: To determine if the new CCs, built excluding mothers with an abnormal weight, are better than standard CCs at identifying SGA. A total of 16,122 neonates were identified as SGA, LGA, or AGA, using the two models. Logistic regression and analysis of covariance were used to calculate the OR and CI for adverse outcomes by group. Gestational age was considered as a covariable. Results: The SGA rates by the new CCs and by the standard CCs were 11.8% and 9.7%, respectively. The SGA rate only by the new CCs was 18% and the SGA rate only by the standard CCs was 0.01%. Compared to AGA by both models, SGA by the new CCs had increased rates of cesarean section, (OR 1.53 (95% CI 1.19, 1.96)), prematurity (OR 2.84 (95% CI 2.09, 3.85)), NICU admission (OR 5.41 (95% CI 3.47, 8.43), and adverse outcomes (OR 1.76 (95% CI 1.06, 2.60). The strength of these associations decreased with gestational age. Conclusion: The use of the new CCs allowed for a more accurate identification of SGA at risk of adverse perinatal outcomes as compared to the standard CCs.

## 1. Introduction

Small for gestational age (SGA) is defined as a birth weight of less than the 10th percentile for gestational age. SGA and neonates are at greater risk of perinatal morbidity and mortality, as well as obesity, cardiovascular diseases, and neurological disorders during childhood and cardiovascular diseases in adult life [1,2,3,4,5].

The effectiveness of SGA screening relies on the accurate estimation of gestational age and fetal weight and depends on the selected reference fetal growth curve. Different standard population birth weight curves are used to differentiate fetuses and newborns with abnormal growth, SGA, and large for gestational age (LGA), from those with normal growth (appropriate for gestational age (AGA)). Standard population fetal growth curves only consider gestational age and sex to calculate weight percentiles [6].

The use of customized fetal and neonatal weight charts allows to differentiate large and small constitutional fetuses from those with pathological intrauterine growth and help in our understanding and diagnosis of abnormal fetal growth [7,8]. The first customized fetal growth charts (CCs) developed by Gardosi et al. [9,10] and adjusted for maternal height, weight, parity, and ethnic origin represent a gold standard for assessing the growth potential of each individual fetus. Different studies have shown that infants classified as SGA according to CCs were at an increased risk of adverse outcomes as compared to infants classified as SGA according to non-customized charts [11,12,13,14,15,16,17,18]. As a result, the use of CCs has expanded worldwide [19].

Abnormal maternal weight (both obesity and underweight) during pregnancy increases oxidative stress, alters the hormonal environment and microbiome, and can modify the epigenetic of the mother–placenta–fetus axis with short- and long-term deleterious consequences for mothers and their offspring [20,21,22,23,24]. Maternal obesity and thinness are also associated with several adverse perinatal outcomes including excessive or restricted fetal growth, preterm delivery, cesarean delivery, gestational diabetes, hypertensive disorders of pregnancy, and infant morbidity and mortality [25,26]. However, to the best of our knowledge, maternal obesity and thinness have never been considered so far as an exclusion criterion in the construction of CCs.

If normal fetal weight curves are calculated excluding the weights of infants born to mothers with obesity or thinness, the efficiency of the models to identify fetuses and newborns with restricted or excessive intrauterine growth will be greater than that of conventional curves, as well as the possibility of avoiding associated adverse perinatal outcomes.

The aim of this study was to establish the effectiveness of a new, customized fetal growth chart (new CC), which was constructed excluding cases with an abnormal maternal pre-pregnancy body mass index (BMI) to identify SGA infants at an increased risk of adverse perinatal outcomes versus that of standard CC

## 2. Material and Method

Maternal characteristics and perinatal outcomes from a sample of 16,122 singleton-pregnancy neonates born at 26–43 weeks of gestation between 2018 and 2020 were collected from the Register of Perinatal Data of the University Hospital Vall d’Hebrón, Barcelona, Spain. From the initial sample of 16,122 infants, 1382 were excluded due to incomplete or implausible data; therefore, the final sample consisted of 14,740 infants.

Newborns were classified by percentile birth weight as large for gestational age (LGA), SGA, or adequate for gestational age (AGA) using a standard customized chart [26] and the new CCs [27], which was constructed excluding cases with abnormal pre-pregnancy maternal BMI (<18.5 or >25 kg/m^2^) [28], only including women with a normal weight.

LGA was defined as a weight above the 90th percentile, SGA as a weight below the 10th percentile, and AGA as a weight between the 10th and 90th percentile.

Factors included in the final stepwise models and their coefficients for standard customized curves [26] and new CCs [27] prediction models for optimal fetal weight are included in Table 1.

### 2.1. Outcomes

The maternal characteristics analyzed were: age, weight, height, BMI, parity, smoking habit, diabetes mellitus, and assisted reproduction techniques required, and the following perinatal outcomes were studied: mode of delivery, gestational age (days), prematurity (less than 28 weeks, between 28 and 34 weeks, and between 34 and 37 weeks of gestational age), birth weight, Apgar score at 1 and 5 min, pH value in the umbilical artery at delivery, admission to the neonatal intensive care unit (NICU), and perinatal mortality (stillbirth and neonatal death). In addition, we considered two composite outcomes (at least one perinatal outcome), 1: cesarean section, shoulder dystocia, Apgar score < 5 at 1st and 5th min, NICU admission or perinatal mortality; and 2: Apgar score < 5 at 1st and 5th min, NICU admission or perinatal mortality. Perinatal outcomes were compared between infants classified as AGA and SGA according to the standard CCs or the new CCs.

### 2.2. Subgroups and Comparisons

In order to assess the effectiveness of the new CCs, the following groups of infants were considered and compared:

- SGA according to the standard CCs, SGA according to the new CCs, SGA according to both the CCs and new CCs, SGA according only to the standard CCs, and SGA according only to the new CCs.
- AGA according only to the new CCs, and AGA according to both the standard CCs and the new CCs.

### 2.3. Statistical Analysis

The distribution of variables was investigated using histograms and the Kolmogorov–Smirnov’s test. Numerical data are reported as mean and standard deviation for parametric variables. Qualitative variables are reported as frequencies and percentages. Differences between groups were studied using the Student’s *t*-test. When three or more groups were compared, homogeneous subsets were indicated at a level of 5%, so that cases that are in the same subset did not differ significantly. Comparison between proportions was completed using the χ^2^ test and Fisher’s exact test when any of the expected values were <5.

Logistic regression was used to calculate the odds ratio (OR) of adverse outcomes taking AGA according to both the CCs and the new CCs as a reference (OR = 1). The confidence intervals for pH were calculated by analysis of covariance. Gestational age was considered as a covariable.

Cohen’s kappa coefficient was used to assess agreement between the chart models and the scale proposed by Landis and Kock [29] was used to describe the level of agreement as follows: 0.21–0.40, “poor”; 0.41–0.60, “moderate”; 0.61–0.80, “good” and 0.81–1.00 “excellent”.

For statistical analysis, SPSS 25.0 software (SPSS Inc., Chicago, IL, USA) was used.

## 3. Results

The 14,740 infants included in the final sample were classified by weight as AGA, SGA, or LGA according to the standard CCs and the new CCs. Maternal characteristics and perinatal outcomes for these groups are shown in Table 2.

**Figure 1:**
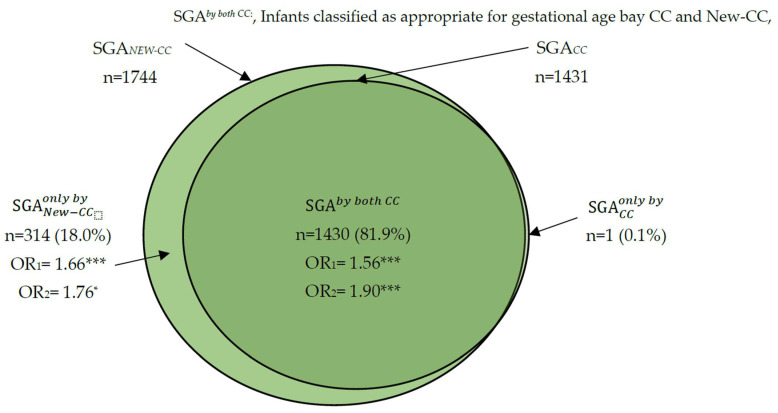
Classification of newborns by birth weight according to the use of conventional customized curves (CCs) and new CCs (new CCs). SGA_CC_ or SGA_NEW CC_, small for gestational age according to the use of conventional curves (CCs) or new, customized curves (new CCs), respectively). ${\mathrm{SGA}}_{CC}^{only\;by}$: Infants classified as SGA according to the CCs and as AGA according to the new CCs. ${\mathrm{SGA}}_{New\;CC_{}}^{only\;by}$: Infants classified as SGA according to the new CCs and AGA according to the CCs. OR_1_: Odds ratio of ${\mathrm{SGA}}_{CC}^{only\;by}$, ${\mathrm{SGA}}_{{}_{NEW}\;CC}^{only\;by}$, and ${\mathrm{SGA}}^{by\;both\;CC}$ versus ${\mathrm{AGA}}^{by\;both\;CC}$ for the composite outcome 1 (cesarean section, Apgar < 7 at 1st min, Apgar < 7 at 5th min, NICU admission or mortality). OR_2_: Odds ratio of ${\mathrm{SGA}}_{CC}^{only\;by}$, ${\mathrm{SGA}}_{New\;CC_{}}^{only\;by}$, and ${\mathrm{SGA}}^{by\;both\;CC}$ versus ${\mathrm{AGA}}^{by\;both\;CC}$ for composite outcome 2 (Apgar < 7 at 1st min, Apgar < 7 at 5th min, NICU admission or mortality).* *p* value < 0.05 and *** *p* value < 0.001.

The rate of SGA by both the conventional CCs and the new CC was 81.9%. The SGA rates according only to the new CCs (SGA according to the new CCs, but AGE according to the standard CC) were 18%, and SGA rates according to only the standard CCs (SGA according to the standard CCs, but AGA according to the new CCs) were less than 0.01% (Figure 1).

The Kappa agreement coefficient between the standard CCs and the new CCs was “excellent” (*Kappa* = 0.906; *p* < 0.001).

Perinatal outcomes were significantly worse in infants classified as SGA according to only the new CCs as compared to infants classified as AGA according to the new CCs; he rate of cesarean section in SGA only according the new CCs was significantly higher than in AGA classified by the new CCs, (31.8% versus 21.4%, *p* < 0.001) and the rate of prematurity was more than double (16.9 % versus 6.9%, *p* < 0.01). The Apgar values at the first and fifth minutes were also significantly higher in SGA only classified by the new CCs (*p* < 0.001 and 0.007, respectively). The rate of NICU admission reached 7.6% in SGA only by the new CCs versus 0.6% in AGA according to the new CC group, (*p* < 0.001). Finally, the rates of composite adverse perinatal outcome 1 and 2 were likewise significantly higher in SGA only according to the new CC group than in AGA by the conventional CCs. Table 3 shows these results.

No differences were found in perinatal outcomes between SGA only in the new CCs and SGA according to the new CCs.

Only one infant was identified as SGA by the standard CCs and AGA by the new CCs (Table 3).

As compared to infants classified as AGA according to both CCs, infants classified as SGA according only to the new CCs had increased rates of cesarean section (OR 1.53 (95% CI 1.19, 1.96)); low Apgar scores (OR 1.81 (95% CI 1.12, 2.93)); prematurity (OR 2.84 (95% CI 2.09, 3.85); NICU admission (OR 5.41 (95% CI 3.47, 8.43)); and composite adverse outcome 1 (OR 1.66 (95% CI 1.30, 2.11)) and composite adverse outcome 2 (OR 1.76 (95% CI 1.06, 2.60)).

The strength of the observed associations decreased significantly with gestational age (Table 4).

## 4. Discussion

Screening for, and adequate management of, fetal growth abnormalities are essential components of antenatal care because growth-related adverse outcomes may be potentially avoidable.

In our study, the rate of SGA was higher according to the new CCs as compared to the standard CCs (11.8% versus 9.7%, respectively).

The use of the new CCs allowed us to identify 18% SGA infants, who according to the conventional CCs, would have been classified as AGA (SGA only according to the new CCs). This rate is very high, and higher than that found by González González et al. [27] in a previous study (2.2%). In their study, the rates of SGA infants according to the new CCs versus the conventional CCs were similar, 14.0% and 13.7%, respectively. In contrast, the rate of infants classified as SGA according to the standard CCs and as AGA according to the new CCs (SGA according only to the standard CCs) was very low (0.01%) and lower than in the González González et al. [27] study (2.2%). Only one infant was identified as SGA by the standard CCs and AGA by the new CCs.

More importantly, in our study, the use of new CCs led to a better identification of SGA with a risk of adverse perinatal outcomes than conventional CCs. Perinatal outcomes were significantly worse in infants classified as SGA according only to the new CCs as compared to infants classified as AGA according to the new CCs. In the SGA only according to the new CC group, the rates of composite adverse perinatal outcome 1 were 37.3% versus 23.5% in AGA according to both the conventional and the new CCs, and the rate of adverse perinatal outcome 2 was multiplied by three. The rate of cesarean section and NICU admission in SGA only according to the new CCs were significantly higher than in AGA by new CCs and the rate of prematurity was more than double.

As well, infants classified as SGA according only to the new CCs showed a higher risk of cesarean section (OR = 1.53), low Apgar test value (OR = 1.81), prematurity (OR = 2.84), NICU admission (OR = 5.41), and composite adverse outcomes 1 and 2 (OR = 1.66 and OR = 1.76, respectively), than infants classified as AGA according to both CCs. These benefits decreased with gestational age.

These results coincide with those of González González et al. [27] and support the importance of constructing customized charts excluding cases with abnormal pre-pregnancy BMI and confirm that the main advantage of the new CCs over conventional CCs is that they allow for a more accurate identification of premature SGA with a risk of adverse perinatal outcomes. The use in clinical practice of new CCs, constructed excluding mothers with pre-pregnancy obesity or thinness, will allow us to identify SGA fetuses and newborns that would have been considered AEG using conventional CCs or non-CCs and offer them specialized care to improves their perinatal outcomes in the short and long term and their health in adult life.

An SGA fetus is one whose growth is below a predefined threshold for its gestational age. SGA fetuses typically have FGR or abdominal circumference below the 10th percentile, although 5th centile, 3rd centile, –2SD, and *Z*-score deviation have also been used as cut-offs in the literature.

There are three fundamental requirements to accurately identify SGA fetuses: precise estimation of gestational age, dating pregnancies by early ultrasound examination at 8–14 weeks, based on measurement of the fetal crown–rump length, is the most reliable method to establish gestational age, accurate calculation of fetal size, ultrasound biometry of the fetus is the gold standard for calculating FGR and assessing fetal growth. The measurements most commonly used are the biparietal diameter, head circumference, abdominal circumference, and femur length. In addition, fetal growth curves as precise as possible to differentiate SGAs at risk of adverse perinatal outcomes. Different reference charts may report different centiles for the same fetal measurement; this may be due to the methodological differences in creating them [30].

Fetal growth depends on several factors, including utero-placental function, maternal disease, maternal cardiovascular function or cardiac disease, maternal nutrition, altitude, smoking and illicit drug use, and the presence of pathological conditions, such as infection, aneuploidy, and some genetic conditions. In CC, the fetal weight and growth are adjusted for variables known to impact fetal size [1,2].

Many authors agree that using standard CCs instead of population curves, adjusted only for the gestational age and gender of the fetus or newborn, allows for identification of an additional group of SGA infants with an increased risk of perinatal morbidity and mortality [11,12,13,14,15,16,17,18,19] although Chiosi et al. [31] were not able to confirm the benefits of standard CCs.

The Royal College of Obstetricians and Gynecologists [32] recommends their use. Guidelines for the monitoring and management of SGA in the Growth Assessment Protocol (GAP) [32,33] combine the use of standard CCs with accreditation training, e-learning support, and audit tools for monitoring SGA. This protocol has led to a year-on-year reduction in stillbirth rates, to their lowest levels ever in the United Kingdom [34]. Customized percentile calculators are freely available via the Gestation Network (www.gestation.net) (accessed on 14 November 2022) that is administered by the Perinatal Institute and have been or are currently in use by clinicians and researchers in more than 30 countries [19].

According to the latest WHO definition, malnutrition refers to deficiencies or excesses in nutrient intake, imbalance of essential nutrients, or impaired nutrient utilization. The double burden of malnutrition consists of both under nutrition and overweight and obesity, as well as diet-related non-communicable diseases [35]. Pre-pregnancy maternal underweight increases the risk of SGA infants, whereas obesity increases the risks of not only LGA infants, but also SGA infants [36,37]. However, to the best of our knowledge, to date, obesity or thinness have been considered as an exclusion criterion in the construction of standard CCs.

In recent years, different fetal growth charts have been developed for international use by Intergrowth-21st [38], the World Health Organization [39], and the National Institute of Child Health and Human Development (NICHD) [40]. They have all been constructed excluding cases with abnormal pre-pregnancy BMI from the calculations. However, the usefulness of these charts in clinical practice has not been proven so far [41,42].

The standard CCs identified more SGA infants at risk of perinatal mortality and morbidity than the population curves and the Intergrowth-21st standard [43,44]. Currently, more than 30 countries worldwide use their own CCs and many others are building their own versions [19,45].

The main strength of our study lies in demonstrating in an external sample, different from that used for the construction of the new CCs, that if mothers with an abnormal pre-pregnancy weight are excluded from the calculations, the efficacy of the CCs to identify the SGA with a risk of adverse perinatal outcomes increases significantly. However, this study is retrospective and the sample analyzed is small. Our results should be confirmed in randomized prospective studies, in different populations, and with larger sample sizes.

## 5. Conclusions

The results obtained in our study support that the new CCs, constructed excluding cases with abnormal pre-pregnancy BMI, identify SGA fetuses and newborns at risk of adverse outcomes more accurately, as compared to standard CCs.

## Figures and Tables

**Table 1:** Coefficients for conventional customized curves [26], and new customized prediction models for optimal weight. Factors included in the final stepwise models [27].

Model		Customized Curves	New Customized Curves _(18.5–25)_
Constant		3289.681	3304.579
Maternal height (MH, cm) (from 163)		9.392	6.987
Maternal weight (MW, kg) (from 65)			
Linear		4.856	7.510
Squared		−0.098	-
Cubed		0.001	-
Parity (Birth ≥ 1)		113.889	113.530
Ethnic origin			
East-Asia		165.560	143.461
South America		161.550	134.161
Rest of Europe		67.927	68.934
North Africa		109.265	62.447
GA (weeks) (from 40)			
Linear		135.413	134.457
Squared		−14.063	−13.435
Cubed		−0.838	−0.803
Sex			
Male		67.188	67.552
Female		−67.188	−67.552
Interactions			
GA (linear) with	Sex	6.890	8.501
	Parity (Birth ≥ 1)	9.032	11.300
	MH (cubed)	0.006	0.008
	MW (squared)	0.260	-
GA (squared) with	MH (linear)	−0.378	-
	MH (squared)	−0.008	−0.011
GA (cubed) with	MH (linear)	−0.032	-
Mean square error		144,630.076	133,659.796
R^2^		0.454	0.451
Coefficient of variation (cv)		0.1156	0.1106

BMI: body mass index; GA: gestational age.

**Table 2:** Maternal characteristics of small, adequate, and large for gestational age infants (SGA, AGA, and LGA) according to the use of new customized curves (new CCs) or conventional customized curves (CC).

	Total (N = 14,740)	New Customized Curves (New CCs)				Conventional Customized Curves (CC)		
		SGA (S) (N = 1744) 11.8%	AGA(A) (N = 10,984) 74.5%	LGA (L) (N = 2012) 13.7%	*p*-Value (Homogeneous Subset)	SGA (N = 1431) 9.7%	AGA (N = 11,197) 76%	LGA (N = 2112) 14.3%
Maternal age, years	31.7 ± 5.9	32.1 ± 5.9	31.7 ± 5.9	31.3 ± 5.9	<0.001 (L; A; S)	32.2 ± 6.0	31.7 ± 5.9	31.4 ± 5.8
Weight, kg	64.4 ± 13.4	66.8 ± 15.8	64.2 ± 13.2	63.3 ± 12.0	<0.001 (L; A; S)	64.9 ± 14.2	64.2 ± 13.2	65.2 ± 13.8
Height, m	161.1 ± 6.6	161.4 ± 6.6	161.2 ± 6.6	160.4 ± 6.9	<0.001 (L; A-S)	161.5 ± 6.5	161.2 ± 6.6	160.4 ± 6.9
BMI, kg/m^2^	24.8 ± 4.9	25.6 ± 5.7	24.7 ± 4.8	24.6 ± 4.3	<0.001 (L-A; S)	24.9 ± 5.1	24.7 ± 4.8	25.3 ± 4.9
Nulliparous	7509 (50.9)	902 (51.7)	5595 (50.9)	1012 (50.3)	0.685	753 (52.6)	5721 (51.1)	1035 (49.0)
Cigarette smoker	2118 (14.5)	459 (26.5)	1511 (13.9)	148 (7.4)	<0.001 (L; A; S)	385 (27.1)	1578 (14.2)	155 (7.4)
Diabetes mellitus	154 (1.0)	12 (0.7)	87 (0.8)	55 (2.7)	<0.001 (S-A; L)	9 (0.6)	87 (0.8)	58 (2.7)
Assisted reproduction techniques	1150 (7.8)	140 (8.0)	863 (7.9)	147 (7.3)	0.333	119 (8.3)	879 (7.9)	152 (7.2)
Gestational age (days)	274 ± 14.2	267 ± 20.4	275 ± 12.6	273 ± 14.6	<0.001 (S; L; A)	267 ± 20.5	275 ± 12.6	273 ± 15.5
Cesarean section	3527 (23.9)	587 (33.7)	2358 (21.5)	582 (28.9)	<0.001 (A; L; S)	488 (34.1)	2406 (21.5)	633 (30.0)
Prematurity	1271 (8.6)	319 (18.3)	753 (6.9)	199 (9.9)	<0.001 (A; L; S)	267 (18.7)	788 (7.0)	216 (10.2)
<28	55 (0.4)	28 (1.6)	18 (0.2)	9 (0.4)		21 (1.5)	18 (0.2)	16 (0.8)
(28–34)	286 (1.9)	105 (6.0)	148 (1.3)	33 (1.6)		89 (6.2)	160 (1.4)	37 (1.8)
(34–37) weeks	930 (6.3)	186 (10.7)	587 (5.3)	157 (7.8)		157 (11.0)	610 (5.4)	163 (7.7)
Umbilical artery pH	7.25 ± 0.094	7.23 ± 0.101	7.25 ± 0.093	7.25 ± 0.088	0.010 (S; A-L)	7.23 ± 0.104	7.25 ± 0.092	7.24 ± 0.094
Apgar < 7at 1st min.	541 (3.7)	151 (10.7)	313 (3.5)	77 (4.7)	<0.001 (A-L; S)	129 (9.0)	325 (2.9)	87 (4.1)
Apgar < 7 at 5th min.	100 (0.7)	36 (2.5)	56 (0.6)	8 (0.5)	<0.001 (L-A; S)	30 (2.1)	57 (0.5)	13 (0.6)
NICU admission	363 (2.5)	140 (8.0)	180 (1.6)	43 (2.1)	<0.001 (L-A; S)	117 (8.2)	192 (1.7)	54 (2.6)
Perinatal mortality	65 (0.4)	28 (1.6)	32 (0.3)	5 (0.2)	<0.001 (L-A; S)	26 (1.8)	31 (0.3)	8 (0.4)
Composite outcome 1	3878 (26.3)	665 (38.1)	2582 (23.5)	631 (31.4)	<0.001 (A; L; S)	549 (38.4)	2642 (23.6)	687 (32.5)
Composite outcome 2	832 (5.6)	245 (14.0)	473 (4.3)	114 (5.7)	<0.001 (A-L; S)	208 (14.5)	495 (4.4)	129 (6.1)

Results are shown as mean ± standard deviation or as frequency (%). Composite outcome 1: cesarean section, Apgar < 7 at 1st min, Apgar < 7 at 5th min, and NICU admission or mortality. Composite outcome 2: Apgar < 7 at 1st min, Apgar < 7 at 5th min, and NICU admission or mortality. S: SGA (small for gestational age); A: AGA (adequate for gestational age); L: LGA (large for gestational age). According to the conventional CC, the rates of SGA, AGA, and LGA were 9.7%, 76.0%, and 14.3%%, respectively. Using the new CC model, the rate of SGA was significantly higher 11.8%, and the rate of LGA and AGA were lower, 13.7% and 74.5%, respectively (*p* < 0.001).

**Table 3:** Perinatal outcomes for adequate and small for gestational age infants (AGA and SGA, respectively) according to the use of new customized curves (new CCs) or conventional customized curves (CCs).

Perinatal Outcome	$AGA_{NewCC}$ (n = 10,984)	$SGA_{NewCC}$ (n = 1744)	$SGA_{CC}^{only\;by}$ (n = 1)	$SGA_{New\;CC_{}}^{only\;by}$ (n = 314; 18%)	*p*-Value			
					P_1_ $SGA_{CC}^{only\;by}$ vs. ${\mathrm{AGA}}_{NewCC_{}}$	P_2_ $SGA_{CC}^{only\;by}$ vs. SGA_*NewCC*_	P_3_ $SGA_{New\;CC_{}}^{only\;by}$ vs. ${\mathrm{AGA}}_{NewCC_{}}$	P_4_ $SGA_{New\;CC_{}}^{only\;by}$ vs. ${\mathrm{SGA}}_{NewCC_{}}$
Cesarean section	2358 (21.5)	587 (33.7)	1 (100)	100 (31.8)	0.215	0.337	<0.001	0.559
Prematurity	753 (6.9)	319 (18.3)	1 (100)	53 (16.9)	0.069	0.183	<0.001	0.578
< 28	18 (0.2)	28 (1.6)	1 (100)	8 (2.5)				
(28–34)	148 (1.3)	105 (6.0)	-	16 (5.1)				
(34–37) weeks	587 (5.3)	186 (10.7)	-	29 (9.2)				
Umbilical artery pH	7.25 ± 0.093	7.23 ± 0.101	-	7.23 ± 0.089	-	-	0.150	0.902
Apgar < 7at 1st min.	313 (3.5)	151 (10.7)	-	22 (9.0)	0.972	0.913	<0.001	0.497
Apgar < 7 at 5th min.	56 (0.6)	36 (2.5)	-	6 (2.4)	0.995	0.979	0.007	0.924
NICU admission	180 (1.6)	140 (8.0)	1 (100)	24 (7.6)	0.016	0.081	<0.001	0.910
Perinatal mortality	32 (0.3)	28 (1.6)	-	2 (0.6)	0.997	0.984	0.244	0.302
- Composite outcome 1	2582 (23.5)	665 (38.1)	1 (100)	117 (37.3)	0.235	0.382	<0.001	0.810
- Composite outcome 2	473 (4.3)	245 (14.0)	1 (100)	38 (12.1)	0.043	0.141	<0.001	0.423

The results are shown as means ± standard deviation or as frequency (%). AGA_*New CCs*_ and SGA_*New CCs*_ = Infants appropriate and small for gestational age according to the new CCs; ${\mathrm{SGA}}_{CC}^{only\;by}$: Infants classified as SGA according to the CCs and as AGA according to the new CCs. ${\mathrm{SGA}}_{New-CC_{}}^{only\;by}$: Infants classified as AGA according to the CCs and as SGA according to the new CCs. NICU = neonatal intensive care unit; vs. = versus. Composite outcome 1: Cesarean section, Apgar < 7 at 1st min, Apgar < 7 at 5th min, and NICU admission or mortality. Composite outcome 2: Apgar < 7 at 1st min, Apgar < 7 at 5th min, and NICU admission or mortality.

**Table 4:** Comparison of the occurrence of perinatal outcomes in the indicated groups with respect to the AGA*^by both CC^* group (infants classified as appropriate for gestational age by both conventional and new customized charts (CCs and new CCs) adjusted by gestational age.

Perinatal Outcome	Small for Gestational Age			$AGA_{}^{by\;both\;CC}$ (N = 10,812)
	$SGA_{}^{by\;both\;CC}$ (N = 1430)	$SGA_{CC}^{only\;by}$ (N = 1)	$SGA_{New\;CC_{}}^{only\;by}$ (N = 314)	
**Cesarean section †**	34.1% 1.55 (1.36, 1.75) ***	100% -	**31.8%** 1.53 (1.19, 1.96) **	21.2%
pH_UA_ (mean ± s.d.; IC_95%_)	7.23 ± 0.10 (−0.022, −0.004) **	-	7.23 ± 0.09; (−0.034, 0.034)	7.25 ± 0.09
pH_UA_ < 7	0.8% 2.41 (1.18, 4.91) *	-	0.3% 1.00 (0.13, 7.43)	0.3%
Apgar < 7 at 1st min. †	9.0% 2.26 (1.79, 2.86) ***	-	**7.0%** 1.81 (1.12, 2.93)*	2.8%
Apgar < 7 at 5th min. †	2.1% 2.43 (1.46, 4.04) **	-	1.9% 2.04 (0.79, 5.24)	0.5%
Prematurity (total) †	18.6% 3.20 (2.74, 3.73) ***	100% -	**16.9%** 2.84 (2.09, 3.85) ***	6.7%
<28	1.4% 17.03 (7.74, 37.46) ***	100% -	**2.6%** 31.38 (12.03, 81.89) ***	0.1%
(28–34)	6.2% 5.06 (3.85, 6.64) ***	-	**5.1%** 4.09 (2.41, 6.95) ***	1.3%
(34–37) weeks	11.0% 2.21 (1.83, 2.66) ***	-	**9.2%** 1.82 (1.23, 2.69) **	5.3%
NICU admission †	8.1% 5.77 (4.52, 7.37) ***	100% -	**7.6%** 5.41 (3.47, 8.43) ***	1.5%
Perinatal mortality †	1.8% 2.95 (1.61, 5.40) ***	-	0.6% 2.38 (0.57, 10.03)	0.3%
Composite outcome (at least one perinatal outcome)				
- Composite outcome 1	38.3% 1.56 (1.38, 1.77) ***	100% -	**37.3%** 1.66 (1.30, 2.11) ***	23.2%
- Composite outcome 2	14.5% 1.90 (1.54, 2.35) ***	100% -	**12.1%** 1.76 (1.06, 2.60) *	4.2%

The data show the percentage and odds ratio (95% confidence interval for the odds ratio) except for the umbilical artery pH. * *p*-value < 0.5; ** *p*-value <0.01; *** *p*-value < 0.001. In bold are the perinatal outcomes that best discriminate the individuals who are classified as large or small for gestational age by only one of the curves. †: The percentages of these perinatal outcomes significantly decrease with increasing gestational age. SGA*^by both CC^*: Infants classified as appropriate for gestational age according to the CCs and new CCs, ${\mathrm{SGA}}_{CC}^{only\;by}$: Infants classified as SGA according to the CCs and as AGA according to the new CCs. ${\mathrm{SGA}}_{New-CC_{}}^{only\;by}$: Infants classified as SGA by the new CCs and AGA according to the CCs. Composite outcome 1: cesarean section, Apgar < 7 at 1st min, Apgar < 7 at 5th min, and NICU admission or mortality. Composite outcome 2: Apgar < 7 at 1st min, Apgar < 7 at 5th min, and NICU admission or mortality.

## Data Availability

If requested, the authors guarantee that the data supporting the results reported in this article may be provided.
